# Entropy Weight Ensemble Framework for Yield Prediction of Winter Wheat Under Different Water Stress Treatments Using Unmanned Aerial Vehicle-Based Multispectral and Thermal Data

**DOI:** 10.3389/fpls.2021.730181

**Published:** 2021-12-20

**Authors:** Shuaipeng Fei, Muhammad Adeel Hassan, Yuntao Ma, Meiyan Shu, Qian Cheng, Zongpeng Li, Zhen Chen, Yonggui Xiao

**Affiliations:** ^1^Institute of Farmland Irrigation, Chinese Academy of Agricultural Sciences, Xinxiang, China; ^2^National Wheat Improvement Center, Institute of Crop Sciences, Chinese Academy of Agricultural Sciences, Beijing, China; ^3^College of Land Science and Technology, China Agricultural University, Beijing, China; ^4^Dezhou Academy of Agricultural Sciences, Dezhou, China

**Keywords:** UAV, multispectral indices, machine learning, remote sensing, thermal infrared, wheat yield

## Abstract

Crop breeding programs generally perform early field assessments of candidate selection based on primary traits such as grain yield (GY). The traditional methods of yield assessment are costly, inefficient, and considered a bottleneck in modern precision agriculture. Recent advances in an unmanned aerial vehicle (UAV) and development of sensors have opened a new avenue for data acquisition cost-effectively and rapidly. We evaluated UAV-based multispectral and thermal images for in-season GY prediction using 30 winter wheat genotypes under 3 water treatments. For this, multispectral vegetation indices (VIs) and normalized relative canopy temperature (NRCT) were calculated and selected by the gray relational analysis (GRA) at each growth stage, i.e., jointing, booting, heading, flowering, grain filling, and maturity to reduce the data dimension. The elastic net regression (ENR) was developed by using selected features as input variables for yield prediction, whereas the entropy weight fusion (EWF) method was used to combine the predicted GY values from multiple growth stages. In our results, the fusion of dual-sensor data showed high yield prediction accuracy [coefficient of determination (*R*^2^) = 0.527–0.667] compared to using a single multispectral sensor (*R*^2^ = 0.130–0.461). Results showed that the grain filling stage was the optimal stage to predict GY with *R*^2^ = 0.667, root mean square error (RMSE) = 0.881 t ha^–1^, relative root-mean-square error (RRMSE) = 15.2%, and mean absolute error (MAE) = 0.721 t ha^–1^. The EWF model outperformed at all the individual growth stages with *R*^2^ varying from 0.677 to 0.729. The best prediction result (*R*^2^ = 0.729, RMSE = 0.831 t ha^–1^, RRMSE = 14.3%, and MAE = 0.684 t ha^–1^) was achieved through combining the predicted values of all growth stages. This study suggests that the fusion of UAV-based multispectral and thermal IR data within an ENR-EWF framework can provide a precise and robust prediction of wheat yield.

## Introduction

Bread wheat is one of the most important food crops that feed 40% of the world population (Liu et al., 2020). The timely and accurate evaluation of the grain yield (GY) before harvest can aid the selection of elite genotypes in large breeding programs (Mcbratney et al., 2005; Panda et al., 2010). Yield advocating traits, such as green biomass, leaf area index (LAI), and chlorophyll contents, have been used for within-season yield prediction (Hassan et al., 2018, 2019a). The canopy temperature is another important indicator of transpiration and leaf water potential under drought and heat stress and can help facilitate the selection of resilient genotypes (Zubler and Yoon, 2020). However, phenotyping most of these traits is destructive, time-consuming, and is associated with a high error probability. Therefore, the nondestructive measurements of the above proxy traits of the GY have been employed to increase the prediction accuracy of crop yield cost-effectively (Yu et al., 2016; Elsayed et al., 2017; Hassan et al., 2019a).

In the past few years, low-altitude remote sensing has attracted interest for its application in high-throughput crop phenotyping (Hassan et al., 2019b; Maimaitijiang et al., 2020). The advances in sensor technology have significantly accelerated the use of unmanned aerial vehicles (UAVs) for data collection with high spectral resolution as compared to satellite platforms (Colomina and Molina, 2014; Sidike et al., 2018). Various types of sensors mounted on UAV platforms, such as multispectral, hyperspectral, RGB, and thermal, are being widely used in the phenotypic evaluation of crops, with satisfactory data accuracy. The UAV-based nondestructive multispectral assessments of the LAI (Comba et al., 2020), biomass (Yue et al., 2019), chlorophyll content (Qiao et al., 2020), nitrogen use efficiency (Yang et al., 2020), senescence (Hassan et al., 2021), and GY (Hassan et al., 2019a) have been reported for several crops. These assessments are based on the spectral reflectance from the canopy of plants in the form of light bands with different wavelengths (Li et al., 2014). Thermal remote sensing is also being applied in precision agriculture to detect water stress (Suyoung et al., 2017) and plant resistance (Ludovisi et al., 2017). Recently, the focus has been increased on combining the data from multiple sources, where a group of datasets from multiple sensors is utilized obtained for plant trait estimation. Multi-source data models have the capability to improve crop trait estimations (Maimaitijiang et al., 2020). The use of canopy temperature and spectral information have been demonstrated to improve the model performance in estimating important plant traits for assessment of biotic/abiotic stress (Appeltans et al., 2020; Zubler and Yoon, 2020) and predicting the yield of soybean (Elmetwalli et al., 2020), barely (Rischbeck et al., 2014), and maize (Zhang et al., 2020). For crop yield prediction, flowering to grain filling stages are highly reliable, with good accuracy and repeatability (Hassan et al., 2019a; Hernandez et al., 2015). The predictions made in most studies have been based on the spectral information of an individual growth stage. The accumulation of VIs from jointing to the grain filling stage using a multiple linear regression algorithm has shown good prediction results in rice (Zhou et al., 2017). Since UAV-based temporal information of multispectral vegetation indices (VIs) and temperature can be obtained cost-effectively from multiple growth stages, combining data across the growth stages could help to achieve higher yield prediction accuracy. Machine learning algorithms have been employed with the canopy spectral features as input to construct models for crop trait evaluation, showing high prediction accuracy and adaptability (Wang et al., 2016; Wang J. et al., 2018). The commonly used machine learning algorithms are the random forest (RF) (Breiman, 2001), support vector machine (SVM) (Sain, 1997), and artificial neural network (ANN) (Bradley, 1995), and these have been successfully used for estimating biomass (Wang et al., 2016), LAI (Wang L. et al., 2018), chlorophyll content (Shah et al., 2019), and water content (Tavakoli and Gebbers, 2019). Among the machine learning algorithms, the emerging elastic net regression (ENR) algorithm has been considered one of the most precise prediction method for regression problems (Hui and Hastie, 2005). The ENR algorithm combines the advantages of ridge regression and least absolute shrinkage and selection operator (LASSO) regression to obtain better prediction results (Ogutu et al., 2012). At present, relatively few studies have been conducted on utilizing information obtained by UAV-based sensors as input to the ENR algorithm for the yield prediction of winter wheat.

The entropy weight algorithm is an emerging method in agricultural studies. It works by allocating the weight-based information entropy of the trait in the model (Li et al., 2011). It has been typically used for feature selection and model combination for combining datasets to assess ecosystem health (Cheng et al., 2020), monitor land-use change (Lu et al., 2014), and evaluate the coverage effectiveness of remote sensing satellites (Li et al., 2018). To the best of our knowledge, the entropy weight method has not been used to predict the yield values from multiple growth stages using UAV datasets. The objectives of this study were (1) to evaluate the potential of UAV-based multispectral and thermal sensors for the yield prediction of wheat using the ENR algorithm, (2) to identify the appropriate wheat growth stage for data collection to maximize the yield prediction accuracy, and (3) to investigate the potential of the entropy weight method in combining the predicted GY values from multiple growth stages.

## Materials and Methods

### Germplasm and Experimental Design

**FIGURE 1:**
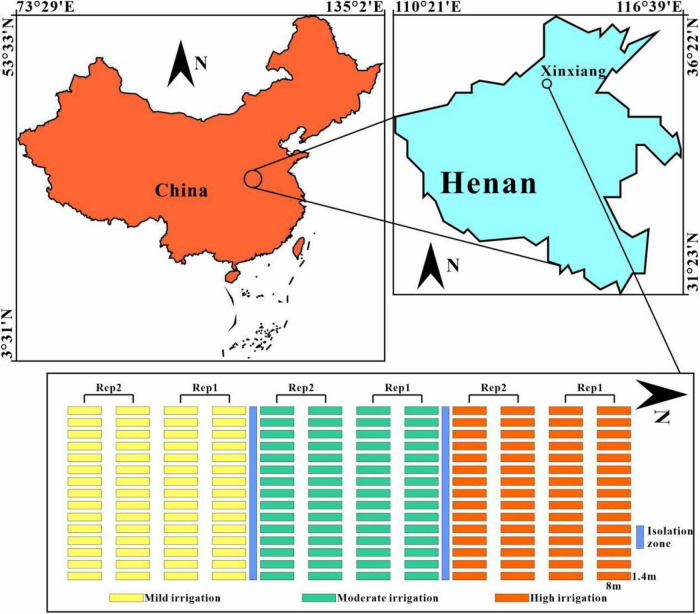
Experimental location, design, and management.

Field trials were conducted at the experimental station of the Institute of Farmland Irrigation of Chinese Academy of Agricultural Sciences in Xinxiang (113.8°E, 35.2°N) during the 2019–2020 cropping season (Figure 1). In total, 30 winter wheat varieties widely cultivated in the Yellow and Huai Valleys Winter Wheat Zone of China were used in this experiment. Germplasm was planted under three water stress treatments, namely, mild irrigation, moderate irrigation, and high irrigation, to obtain the UAV-based multispectral, thermal, and ground-truth GY data. Irrigation for each treatment was performed in the tillering, wintering, reviving, jointing, heading, and grain filling stages using a laterally moving sprinkler irrigation machine. The irrigation volume was calculated by the flow rate of the sprinkler nozzle and the duration of irrigation. The total irrigation volume for the mild, moderate, and high irrigation treatments were 145, 190, and 240 mm, respectively (Table 1). A completely randomized block design with two replications was adopted for the experiment. The size of each plot was maintained at 11.2 m^2^ with the dimensions of 8 m × 1.4 m, representing one cultivar with six rows at a spacing of 0.20 m. Field management (e.g., disease and pest control, fertilizer) was maintained at optimal levels depending on the local conditions. In the 2019–2020 growing season, the total precipitation was 115 mm, and the monthly average temperature was highest (23°C) in July and lowest (−6°C) in January. Wheat was harvested using a plot combine harvester in June 2020. The GY of each plot was weighed at a moisture content of approximately 12.5%.

**TABLE 1:** Irrigation strategy for each treatment.

Growth stage	Mild irrigation (mm)	Moderate irrigation (mm)	High irrigation (mm)
Tillering	35	35	35
Wintering	35	35	35
Turning green	20	25	35
Jointing	20	35	50
Heading	20	35	50
Grain filling	15	25	35
Total	145	190	240

### Data Acquisition and Processing

**FIGURE 2:**
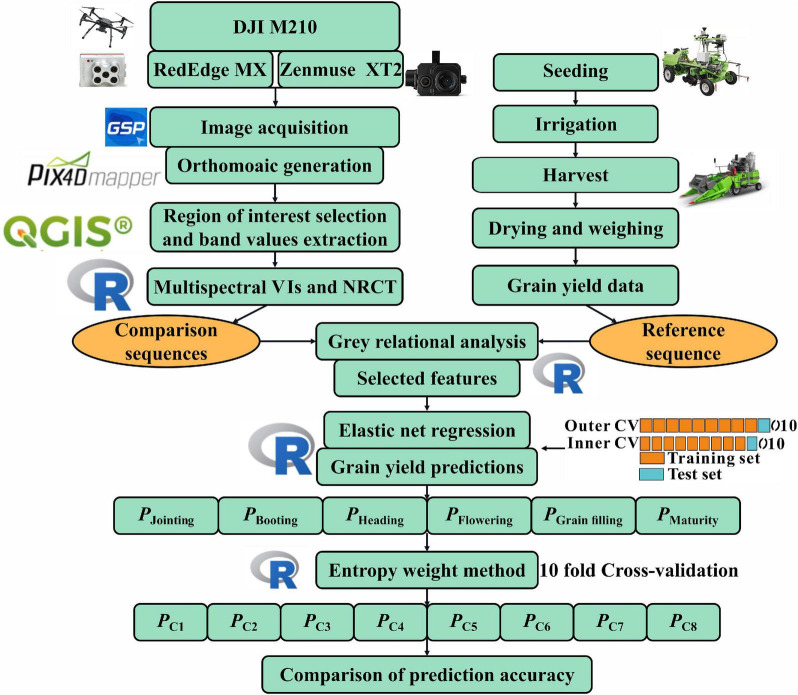
Schematic workflow of the methodology used in this study. *P* denotes the predicted grain yield (GY) value, and C1–C8 indicate the combinations of the values predicted from multiple growth stages. CV, cross-validation; VIs, vegetation indices; NRCT, normalized relative canopy temperature.

Figure 2 shows the workflow for the data acquisition. A DJI M210 (DJI Technology Co., Shenzhen, China) carrying a RedEdge MX (MicaSense Parrot, France) multispectral camera and a Zenmuse XT2 (DJI Technology Co., Shenzhen, China) thermal sensor was used to collect high-resolution multispectral and thermal images simultaneously. The RedEdge MX featured five spectral sensors, namely, blue (475 nm), green (560 nm), red (668 nm), red-edge (717 nm), and near-IR (842 nm). The RedEdge MX camera automatically adjusts the ambient light effects through the sunshine sensor, thereby minimizing the error in the multispectral images. Zenmuse XT2 contains an 8-mm lens with a 57.12° × 42.44° field of view to record temperature measurements in the 7.5–13.5-μm spectral range with a measurement accuracy of ± 5°C. The DJI ground station was used as an automated flight control system, allowing the user to define the air route and customize the mission plan. Flight mission was executed for all the six growth stages from 11 a.m. to 1 p.m. under a cloudless sky. To avoid the effect of the phenological differences between treatments, the flight missions for each treatment were collected according to the growth stages. To obtain high-resolution images, each flight was set at an altitude of 30 m with 85% front and 80% side image overlapping. Before and after each flight, the calibration board was photographed to convert the digital number (DN) value of the multispectral image into reflectance during subsequent data processing. During the flights, the surface temperature of 12 boards was measured using a handheld thermometer for the radiometric calibration of the thermal images. To obtain the geographic reference of the multisensor UAV image, 18 ground control points (GCPs) were evenly arranged in the field, and their coordinates were measured with a millimeter-level accuracy using a differential global positioning system.

The Pix4Dmapper software (Pix4D SA, Lausanne, Switzerland) was employed for the orthomosaic generation using the UAV-based multispectral and thermal IR images. The geographic coordinates (World Geodetic System, 1984) of the GCPs were used in the photogrammetric workflow of Pix4Dmapper to improve the accuracy of the composite orthomosaics. Dense point clouds were generated using the structure-from-motion (SfM) method in Pix4Dmapper along with the photogrammetric workflow. After radiometric correction, the DN values of the multispectral and thermal IR images were converted to reflectance and temperature (°C). To extract the reflectance and temperature information for each plot, the orthomosaic images were segmented into 180 polygon shapes with assigned IDs defining the cultivars under different irrigation treatments. Polygon shape generation and information extraction are completed in QGIS 3.1.0.^1^ A total of 22 indices were used in this study, of which 21 VIs were estimated from multispectral reflectance, and 1 index was calculated from the canopy temperature across the irrigation treatments (Table 2).

**TABLE 2:** Formulae of multispectral vegetation indices and normalized relative canopy temperature.

Acronym	Index	Formulae	Developer(s)
CIRE	Chlorophyll index RedEdge	(R_NIR_/R_RE_)−1	Gitelson et al., 2003
DVI	Difference vegetation index	R_NIR_−R_R_	Tucker et al., 1979
EVI	Enhanced vegetation index	2.5×(R_NIR_−R_R_)/(1R_NIR_ + 6×R_R_−7.5×R_B_)	Huete et al., 2002
GNDVI	Green normalized difference vegetation index	(R_NIR_−R_G_)/(R_NIR_ + R_G_)	Gitelson et al., 1996
MCARI1	Modified chlorophyll absorption in reflectance index 1	(R_REG_−R_R_−0.2×((R_REG_−R_G_))×(R_REG_/R_R_)	Daughtry et al., 2000
PSRI	Plant senescence reflectance index	(R_R_−R_B_)/R_NIR_	Merzlyak et al., 1999
MSR	Modified simple ratio index	$\left(\left({\text{R}}_{\text{NIR}}/{\text{R}}_{\text{R}}\right)-1\right)/\sqrt{{\text{R}}_{\text{NIR}}/{\text{R}}_{\text{R}}1}$	Chen, 1996
MTCI	MERIS terrestrial chlorophyll index	(R_NIR_−R_REG_)/(R_RE_−R_R_)	Dash and Curran, 2004
MTVI2	Modified triangular vegetation index 2	$1.5\times \left[1.2\times \left({\text{R}}_{\text{NIR}}-{\text{R}}_{\text{G}}\right)-2.5\times \left({\text{R}}_{\text{R}}-{\text{R}}_{\text{G}}\right)\right]/CPSTABLEENTER\left[\sqrt{2\times {\left({\text{R}}_{\text{NIR}}+1\right)}^{2}-6\times {\text{R}}_{\text{NIR}}+5\times \sqrt{{\text{R}}_{\text{R}}}}-0.5\right]$	Haboudane et al., 2004
NDVI	Normalized difference vegetation index	(R_NIR_−R_R_)/(R_NIR_ + R_R_)	Rouse, 1972
NDVIRE	Normalized difference vegetation index RedEdge	(R_NIR_−R_REG_)/(R_NIR_ + R_REG_)	Elsayed et al., 2015
NLI	Nonlinear vegetation index	(R_NIR_×R_NIR_−R_R_)/(R_NIR_×R_NIR_ + R_R_)	Goel and Qin, 1994
OSAVI	Optimized soil-adjusted vegetation index	(R_NIR_−R_R_)/(R_NIR_ + R_R_ + 1.6)×1.16	Rondeaux et al., 1996
PPR	Plant pigment ratio	(R_G_−R_B_)/(R_G_ + R_B_)	Metternicht, 2003
RDVI	Re-normalized difference vegetation index	$\left({\text{R}}_{\text{NIR}}-{\text{R}}_{\text{R}}\right)/\sqrt{\left({\text{R}}_{\text{NIR}}+{\text{R}}_{\text{R}}\right)}$	Wang et al., 1998
RVI	Ratio vegetation index	(R_NIR_/R_R_)	Ba Ret and Guyot, 1991
NRI	Nitrogen reflectance index	(R_G_−R_R_)/(R_G_ + R_R_)	Diker and Bausch, 2003
SAVI	Soil-adjusted vegetation index	(R_NIR_−R_R_)/(R_NIR_ + R_R_ + 0.5)×1.5	Huete, 1988
SIPI	Structure insensitive pigment index	(R_NIR_−R_B_)/(R_NIR_ + R_B_)	Penuelas et al., 1995
TCARI	Transformed chlorophyll absorption ratio index	3[(R_REG_−R_R_)−0.2×(R_REG_−R_G_)×R_REG_/R_R_]	Eitel et al., 2007
TVI	Triangular vegetation index	0.5×[120×(R_NIR_−R_G_)−200×(R_R_−R_G_)]	Broge and Leblanc, 2001
NRCT	Normalized relative canopy temperature	$\frac{T_{i}-T_{min}}{T_{max}-T_{min}}$	Elsayed et al., 2015

*R_B_, R_G_, R_R_, R_REG_, and R_NIR_ represent the reflectance of the blue, green, red, red-edge, and near-IR bands of RedEdge MX, respectively. T represents the canopy temperature obtained from Zenmuse XT2.*

### Gray Relational Analysis

In a gray relational analysis (GRA), a system with incomplete information is called a gray system, meaning that the relationship between the factors is uncertain (Aslan et al., 2012). When the experiment is unclear or when the experimental method cannot be implemented accurately, a gray analysis can help overcome the drawbacks in statistical regression (Jin et al., 2013). For example, there is a close relationship between VIs and yield; however, their detailed relationships remain unclear. Therefore, the main purpose of the GRA is to measure the degree of relationship within this system by analyzing the gray relationships between VIs and GY. The GRA procedure includes the following steps:

1. The reference series reflect the characteristics of the system behavior, and the comparison series influences the system behavior. In this study, the GY was considered the reference series, and each index was considered a comparison series. The reference sequence is represented by the following formula:

(1) $$X_{0}=X_{0}\left(k\right)|k=1,2\ldots n$$

where *n* represents the number of samples, and *n* is 180 in this study. Comparison data series can be expressed as follows:


(2) $$X_{i}=X_{i}\left(k\right)|k=1,2\ldots n,i=1,2\ldots m$$

There are *m* comparison data series, each containing *n*-values.

2. Data in each factor column in the system may have different dimensions, making it difficult to compare or obtain a correct conclusion when comparing. Therefore, to ensure the reliability of the results, the following non-dimensional processing of the data is generally required when performing the GRA:

(3) $$x_{i}\left(k\right)=\frac{X_{i}\left(k\right)}{X_{i}\left(l\right)}$$

(4) $$X_{i}\left(l\right)=\frac{1}{n}\sum_{k=1}^{n}X_{i}\left(k\right)$$

3. The calculation of the difference data series △_*i*_ is as follows:

(5) $${△}_{i}=\left(\left|x_{01,}-x_{i1}\right|,\left|x_{02,}-x_{i2}\right|,\ldots \left|x_{0n,}-x_{in}\right|\right)$$

4. The gray relational coefficient *ξ_*i*_*(*k*) for the *k*th data point in the *i*th difference data series can be expressed as follows:

(6) $$\xi_{i}\left(k\right)=\frac{{△}_{\text{min}}+\zeta {△}_{\text{max}}}{{△}_{i\left(k\right)}+\zeta {△}_{\text{max}}}$$

where △_min_ and △_max_ are the global maximum and minimum values in the difference data series, respectively. △_*i*(*k*)_ is the *k*th value in the △_*i*_ difference data series, and ζ is the distinguishing coefficient: ζ∈ [0, 1]. In this study, the distinguishing coefficient is set to 0.5.

5. Generally, the average value of the gray relational coefficient is taken as the gray relational degree (GRD), which is expressed as follows:

(7) $$\gamma_{i}=\frac{1}{n}\sum_{k=1}^{n}\xi_{i}\left(K\right).$$

### Elastic Net Regression

To avoid the instability of the LASSO solutions when the input features are highly correlated (e.g., a large number of VIs constructed from limited bands), the ENR has been proposed to analyze the high-dimensional data. The ENR is an extension of the LASSO, which is robust to severe multicollinearity among the input features (Ogutu et al., 2012). The ENR combines the penalties of the ridge regression (ℓ_1_) and LASSO (ℓ_2_) and can be expressed as follows:


(8) β^(enet)=(1+λ2n){|βargmin|y-Xβ||+22λ2||β||+22λ1||β||}1

On setting α = *λ*_2_/(*λ*_1_ + *λ*_2_), the ENR is equivalent to the minimizer of the following:


(9) β^(enet2)=|βargmin|y-Xβ||22

subject to Pα(β)=(1-α)||β||1+α||β||≤22sforsomes, where *P*_α_(β) represents the penalty of the ENR. The ENR can be considered LASSO and ridge regression when *a* = 0 and 1, respectively. The ℓ1 part of the ENR is used for automatic variable selection, while the ℓ2 part encourages grouped selection and stabilizes the solution paths with respect to the random sampling, thereby improving the prediction results. By introducing a grouping effect when selecting the variable, a group of highly correlated input features tends to have similar coefficients. The ENR can choose the groups of correlated features when these groups are unknown in advance. Notably, the ENR selects more than *n* variables when *p*>> *n*, which is different from the LASSO. In this study, there is inevitably a high correlation between the various VIs. Therefore, the ENR will be an ideal choice when using VIs as the input features for yield prediction.

### Modeling Framework

In this study, a 10-fold outer cross-validation method was adopted to train and test the model. To avoid contingency, we conducted 50 iterations for the outer cross-validation, resulting in a total of 500 models. The average of the accuracy evaluation index generated from the 500 models was used to evaluate the model performance. In the process of outer cross-validation, the inner cross-validation and the grid search were conducted for parameter tuning of the ENR models (Figure 2). In the outer cross-validation, the VIs and GY data were randomly divided into 10 equal subsets. One of them was used for testing each time, and the remaining nine subsets were used for training. Each training set of the outer cross-validation was evenly divided into 10 sets, similar to the outer cross-validation. One of them was used for testing, and the nine subsets were used for training. During the inner cross-validation process, multiple combinations of the candidate parameters were set in the inner training set for model construction and then tested on the inner test set. Each parameter combination was tested 10 times, and the hyperparameter combination with the lowest average test error was set for the outer cross-validation for model training. This study uses the R package ‘‘caret’’^2^ to construct the ENR model for yield prediction. In the “caret” package, the parameters to be adjusted are the *fraction* and quadratic penalty parameter *lambda*. Table 3 represents the candidate ranges of these two parameters.

**TABLE 3:** Candidate hyperparameters for elastic net regression.

Number	*Lambda*	*Fraction*	Number	*Lambda*	*Fraction*
1	0.050	0.000E+00	16	0.541	3.162E-03
2	0.083	1.000E-04	17	0.574	4.047E-03
3	0.116	1.280E-04	18	0.607	5.179E-03
4	0.148	1.638E-04	19	0.640	6.629E-03
5	0.181	2.096E-04	20	0.672	8.483E-03
6	0.214	2.683E-04	21	0.705	1.086E-02
7	0.247	3.433E-04	22	0.738	1.389E-02
8	0.279	4.394E-04	23	0.771	1.778E-02
9	0.312	5.623E-04	24	0.803	2.276E-02
10	0.345	7.197E-04	25	0.836	2.913E-02
11	0.378	9.211E-04	26	0.869	3.728E-02
12	0.410	1.179E-03	27	0.902	4.771E-02
13	0.443	1.509E-03	28	0.934	6.105E-02
14	0.476	1.931E-03	29	0.967	7.814E-02
15	0.509	2.471E-03	30	1.000	1.000E-01

Moreover, we tested the model performance on the test samples in the cross-validation procedure to test the adaptability of the model. The coefficient of determination (*R*^2^), root mean square error (RMSE), relative root-mean-square error (RRMSE), and mean absolute error (MAE) were adopted to evaluate the model performance. The calculation formulae of the parameters are as follows:


(10) $$R^{2}=1-\frac{\sum_{i=1}^{n}{\left(y_{i}-{\hat{y}}_{i}\right)}^{2}}{\sum_{i=1}^{n}{\left(y_{i}-\bar{y}\right)}^{2}}$$

(11) $$\text{RMSE}=\sqrt{\frac{1}{n}\sum_{i=1}^{n}{\left(y_{i}-{\hat{y}}_{i}\right)}^{2}}$$

(12) $$\text{RRMSE}={\frac{\text{RMSE}}{\bar{y}}}^{*}100\%$$

(13) $$\text{MAE}=\frac{1}{n}\sum_{i=1}^{n}\left|y_{i}-{\hat{y}}_{i}\right|$$

where *n* represents the number of samples, *y_i_* and ${\hat{y}}_{i}$ are the measured and predicted GY of sample *i*, and $\bar{y}$ is the average value of the measured GY. The higher the *R*^2^-value, the lower the RMSE, RRMSE, and MAE values and the better the performance of the model for GY prediction.

### Entropy Weight Method

The ENR algorithm was independently implemented at each growth stage. Instead of using these results to predict the GY individually, we proposed an entropy weight fusion (EWF) model that combines the predicted results from the different growth stages *via* weights obtained during the model training stage. The basic mechanism of the entropy weight method is to use the entropy to characterize the degree of disorder in the system (Farhadinia, 2017). In this method, the relative error between the predicted and measured values of the GY obtained in an individual growth stage by the selected *i*th prediction model can be expressed as follows:


(14) Eij{1,when|(yj-yij)yj|≥1;|(yj-yij)yj|,when 0≤|(yj-yij)yj|≤1

where *i* = (1, 2, 3…*m*), *j* = (1, 2, 3…*n*), and *y*_*ij*_ represents the predicted value of the yield forecast model for the *i*th individual growth stage on the *j*th plot. The process for calculating the weights is as follows:

The relative error ratio was calculated between the predicted value of the *i*th individual growth stage and the measured value at plot *j*:


(15) $$P_{ij}=\frac{E_{ij}}{\sum_{i=1}^{n}E_{ij}}$$

where $\sum_{i=1}^{n}P_{ij}=1$. The entropy value *h*_*i*_ was calculated for the relative error in the *i*th individual growth stage prediction:


(16) $$h_{i}=-k\sum_{j=1}^{n}\left[P_{ij}\text{ln}\left(P_{ij}\right)\right]k=1/\text{ln}\left(n\right)$$

The relative error variation coefficient was determined based on the principle of the opposite of the entropy value and its degree of variation:


(17) $$d_{i}=1-h_{i}$$

The weight was then obtained for the predicted output value from a single growth stage:


(18) $$w_{i}=\frac{1}{n}\left(1-d_{i}/\sum_{i=1}^{n}d_{i}\right)$$

The weights were obtained by combining the output forecast values from the multiple growth stages. The final output forecast value can be expressed as follows:


(19) $$\hat{y}=\sum_{i=1}^{n}w_{i}y_{ij}$$

The higher the entropy of the prediction error sequence of a single growth stage, the lower the degree of variation and the greater the weight. The entropy weight method fully considers the relative error in the output prediction value from the different growth stages. Therefore, the predicted results from the multiple growth stages complement each other to improve the accuracy of yield prediction. In this study, eight combinations were created to evaluate the accuracy of the entropy weight method for GY prediction. Table 4 represents a detailed description of each combination.

**TABLE 4:** Combination of different growth stages used in the model for grain yield prediction.

Combination	Combination of growth stages
C1	Jointing, booting, heading
C2	Jointing, booting, heading, flowering
C3	Jointing, booting, heading, flowering, grain filling
C4	Jointing, booting, heading, flowering, grain filling, maturity
C5	Booting, heading, flowering
C6	Heading, flowering, grain filling
C7	Flowering, grain filling, maturity
C8	Heading, flowering, maturity

## Results

### Descriptive Statistics of Grain Yield

**FIGURE 3:**
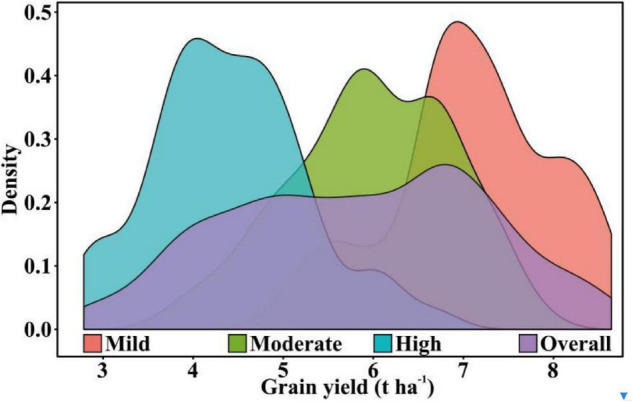
Grain yield distribution curves under various irrigation treatments.

The distribution of yield from wheat plots is shown in Figure 3. The GY was normally distributed under all the irrigation treatments as well as across the treatments. The GY was found to be higher under high irrigation treatments than under the moderate and mild irrigation treatments. The mean GY values for the high, moderate, and mild irrigation treatments were 7.09, 5.99, and 4.40 t ha^–1^, respectively. The highest coefficient of variation (19.51%) was observed in the mild irrigation treatment and the lowest (12.70%) in the high irrigation treatment. The overall range of the GY data across the irrigation treatment was 2.79–8.64 t ha^–1^, with a data variation of up to 24.35%. Across treatment data with this type of variation can help evaluate the prediction accuracy of the model.

### Results of Gray Relational Analysis and Feature Selection

A total of 22 indices were ranked using the GRA method. Table 5 lists the results for all the growth stages. The GRD of the normalized relative canopy temperature (NRCT) ranked first for the jointing, booting, and flowering stages and relatively high for the heading (rank = 10) and grain filling (rank = 9) stages. However, the NRCT ranked last at maturity. The rankings for most VIs were unstable across all the growth stages. For example, plant pigment ratio (PPR) and difference vegetation index (DVI) had a high ranking at both jointing and booting but ranked low at flowering and grain filling. In accordance with the GRA mechanism, the higher the GRD between the main and the reference sequence, the more closely the sequences are related, which indicates a close relationship between the NRCT and the yield during the multiple growth stages.

**TABLE 5:** Ranking of indices using the gray relational analysis (GRA) for six growth stages.

Rank	Jointing	Booting	Heading	Flowering	Grain filling	Maturity
1	NRCT	NRCT	NLI	NRCT	MSR	TVI
2	PPR	PPR	NDVI	RVI	RVI	NLI
3	OSAVI	SAVI	TCARI	MSR	NDVI	SAVI
4	MTVI2	OSAVI	MSR	NDVI	NLI	RDVI
5	SAVI	RDVI	RVI	NLI	NRI	EVI
6	RDVI	MTVI2	MTVI2	OSAVI	OSAVI	DVI
7	MCARI	PSRI	OSAVI	MTVI2	MTVI2	OSAVI
8	PSRI	TCARI	PPR	TCARI	CIRE	MTVI2
9	TCARI	DVI	SAVI	RDVI	NRCT	PSRI
10	DVI	TVI	NRCT	NRI	NDVIRE	MCARI
11	TVI	MCARI	SIPI	SAVI	GNDVI	MTCI
12	EVI	EVI	RDVI	GNDVI	RDVI	NDVI
13	SIPI	SIPI	MCARI	SIPI	SIPI	RVI
14	GNDVI	NLI	PSRI	PSRI	PSRI	MSR
15	NRI	NDVI	EVI	EVI	SAVI	CIRE
16	NDVIRE	MSR	DVI	CIRE	EVI	NDVIRE
17	NLI	RVI	TVI	NDVIRE	TCARI	SIPI
18	CIRE	MTCI	GNDVI	DVI	TVI	NRI
19	MTCI	CIRE	NRI	TVI	DVI	GNDVI
20	NDVI	NRI	NDVIRE	MTCI	MTCI	TCARI
21	MSR	GNDVI	CIRE	PPR	MCARI	PPR
22	RVI	NDVIRE	MTCI	MCARI	PPR	NRCT

**FIGURE 4:**
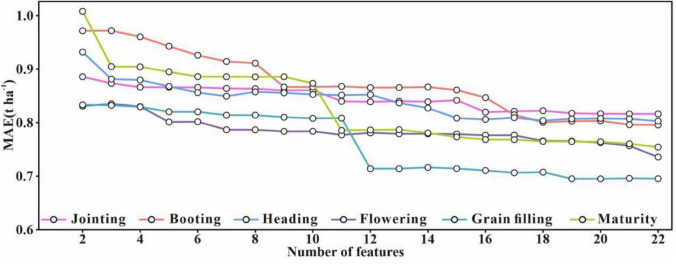
Model training error as a function of the number of features. The order of input of features depends on the gray relational degree (GRD). MAE, mean absolute error.

To further explore the features with better performance and to reduce the dimensionality of the data, the top feature was iteratively added into the ENR. The performance of the model (i.e., MAE) in the training process was updated until all the features were inputted into the ENR (Figure 4). Among the six developmental stages, the grain filling stage yielded the lowest error, and it tended to be stable when the number of features was 19. The highest error was observed in jointing, and the model showed a stable tendency after inputting 16 features. The appropriate numbers of input features for the booting, heading, flowering, and maturity stages were found to be 18, 18, 22, and 22, respectively.

### Performance of Elastic Net Regression Model for Individual Growth Stage

**FIGURE 5:**
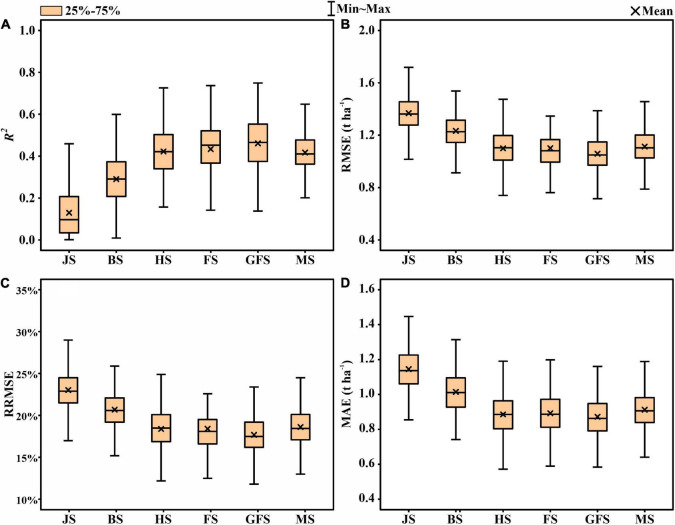
Statistical distributions of **(A)** coefficient of determination (*R*^2^), **(B)** root mean square error (RMSE), **(C)** relative root-mean-square error (RRMSE), and **(D)** mean absolute error (MAE) of the elastic net regression (ENR) algorithm for GY prediction using multispectral features in test phases. JS, jointing stage; BS, booting stage; HS, heading stage; FS, flowering stage; GFS, grain filling stage; MS, maturity stage.

**FIGURE 6:**
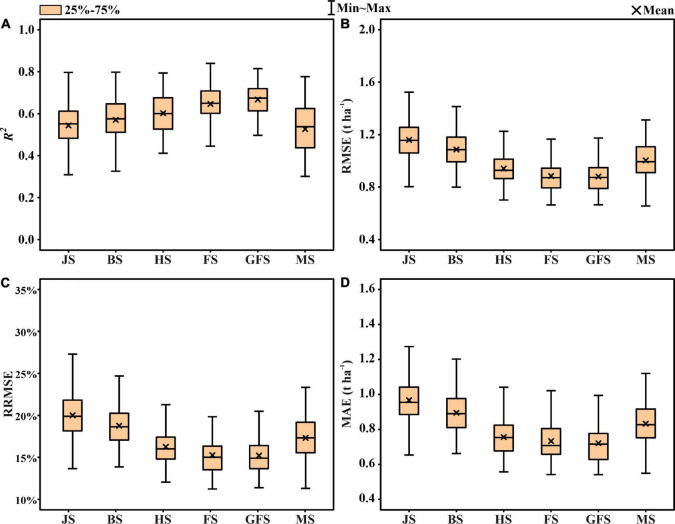
Statistical distributions of **(A)** *R*^2^, **(B)** RMSE, **(C)** RRMSE, and **(D)** MAE of the ENR for GY prediction using both multispectral and thermal features in test phases. JS, jointing stage; BS, booting stage; HS, heading stage; FS, flowering stage; GFS, grain filling stage; MS, maturity stage.

To analyze the improvement of the thermal data for yield prediction accuracy, the model was first built using the features extracted from the multispectral images (Figure 5). The mean prediction values for the grain filling stage was *R*^2^ = 0.461, followed by flowering (*R*^2^ = 0.432), heading (*R*^2^ = 0.422), maturity (*R*^2^ = 0.417), booting (*R*^2^ = 0.290), and jointing (*R*^2^ = 0.130). Figure 6 represents the accuracy assessment results of the ENR model for GY predictions by using both thermal and multispectral features. The results show that the dual-sensor data fusion method achieves higher prediction accuracy at all measured stages compared to using single multispectral sensor-based features. As with using only multispectral features, the ENR showed the highest prediction results with a low error at the grain filling (*R*^2^ = 0.667) stage. The mean prediction results at jointing, booting, heading, flowering, and maturity were *R*^2^ = 0.544, *R*^2^ = 0.571, *R*^2^ = 0.602, *R*^2^ = 0.640, and *R*^2^ = 0.527, respectively.

**FIGURE 7:**
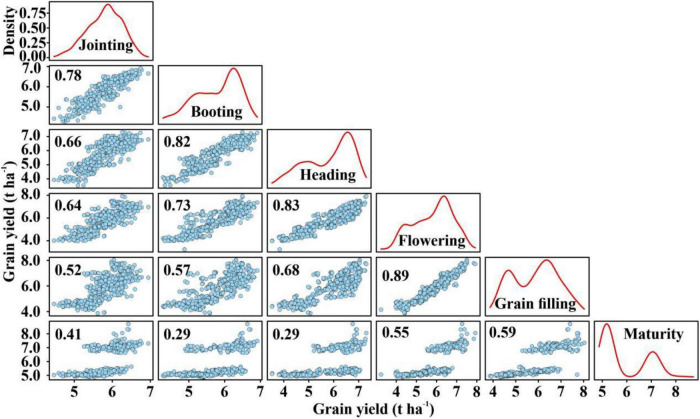
Regression plots, density curve, and *R*^2^-values between predicted GY in six developmental stages.

After obtaining the predicted GY using thermal IR and multispectral features, the regression between the predicted GY from the various stages was conducted (Figure 7). High correlations ranging from *R*^2^ = 0.59 to *R*^2^ = 0.89 between adjacent growth stages were observed across the growth stages. Moreover, the greater the interval between the growth stages, the lower the *R*^2^-value. For example, the *R*^2^ between the jointing stage and the booting, heading, flowering, grain filling, and maturity stages were 0.78, 0.66, 0.64, 0.52, and 0.41, respectively. In comparison, the correlations between the predicted yield in the maturity and other growth stages were lower, with quite weak regression values ranging from *R*^2^ = 0.41 to *R*^2^ = 0.59. There were differences in the distribution curves of predicted GY values, which provides complementary information.

### Performance of Entropy Weight Fusion Method

**FIGURE 8:**
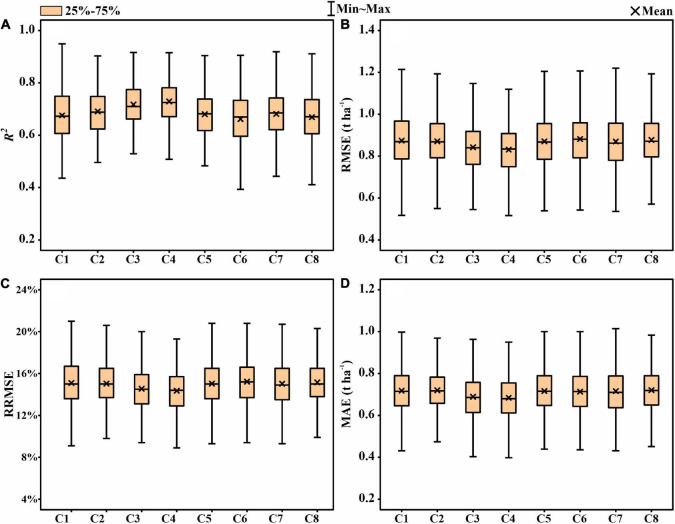
Statistical distributions of **(A)** *R*^2^, **(B)** RMSE, **(C)** RRMSE, and **(D)** MAE of the ENR model that uses both multispectral and thermal features from different stages as inputs.

For comparison with the EWF method, multispectral and thermal features from multiple stages were used as the inputs of ENR to the training model. The results indicated that combining the features of multiple stages increases the accuracy of yield prediction than individual stages (Figure 8). The C4 yielded the highest *R*^2^-value of 0.725, followed by C3 (*R*^2^ = 0.717) and C2 (*R*^2^ = 0.691). The remaining combinations achieved similar prediction accuracy (*R*^2^ = 0.669–0.681). However, the obvious fluctuations of accuracy parameters (*R*^2^, RMSE, RRMSE, and MAE) with wide ranges were observed.

**FIGURE 9:**
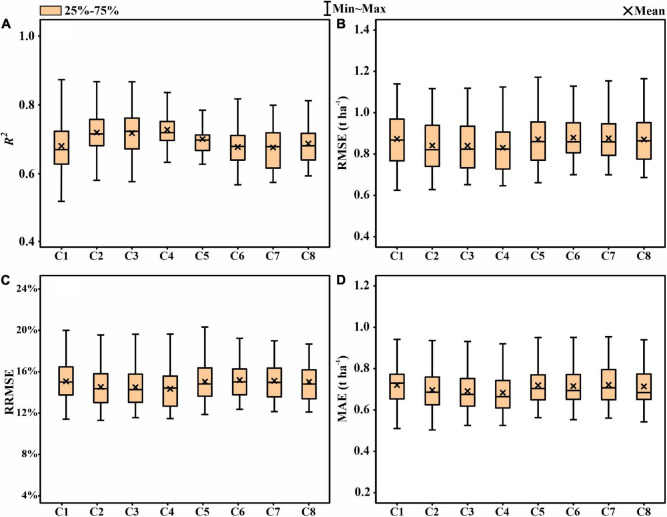
Statistical distributions of **(A)** *R*^2^, **(B)** RMSE, **(C)** RRMSE, and **(D)** MAE of the entropy weight fusion (EWF) method for GY prediction in the test phases.

**FIGURE 10:**
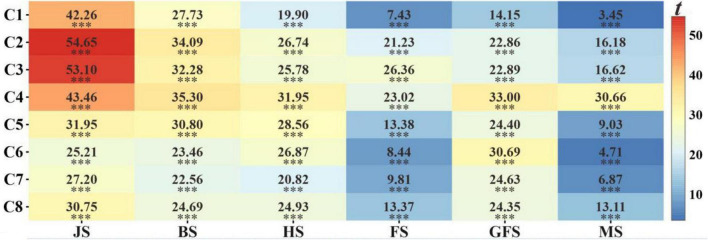
Results from paired *t*-test between model *R*^2^ obtained from the EWF method and the individual stages. ^***^ significant at *P* ≤ 0.001; JS, jointing stage; BS, booting stage; HS, heading stage; FS, flowering stage; GFS, grain filling stage; MS, maturity stage.

Figure 9 represents the performance of the EWF model in predicting the GY using the combined predicted values from the multiple growth stages. Comparing with the individual growth stages, the EWF model also provides a more accurate result regardless of the number of stages adopted. Among the eight combinations, the optimal test results of the EWF model were observed in C4, with a mean *R*^2^ of 0.729. An increase of 0.062 compared with the highest mean *R*^2^-value was observed in the grain filling stage (*R*^2^ = 0.667). Moreover, the RMSE, RRMSE, and MAE values were reduced to 0.831 t ha^–1^, 14.3%, and 0.684 t ha^–1^, respectively. A low prediction was observed in C1 (*R*^2^ = 0.681). Compared to C5 (*R*^2^ = 0.692), C6 (*R*^2^ = 0.678), C7 (*R*^2^ = 0.677), and C8 (*R*^2^ = 0.688), C2 (*R*^2^ = 0.721) and C3 (*R*^2^ = 0.719) had a more accurate predictions. The fluctuations in the accuracy parameters (*R*^2^, RMSE, RRMSE, and MAE) of the EWF model were more moderate compared to the model that directly used multistage features as inputs (Figures 8, 9), which again demonstrates the stability of the EWF method. A paired *t*-test was utilized to assess whether the EWF models performed statistically high in terms of the *R*^2^-values compared with the other models (Figure 10). The results showed significantly high *R*^2^-values for the EWF model in all the growth stage combinations.

## Discussion

The UAV-based phenotyping is an emerging technique in practical crop breeding. Previous studies have shown that the UAV-based features and the machine learning model can be used together to predict crop yields in breeding work with a large number of crop genotypes (Osval et al., 2017; Fei et al., 2021). In this study, ENR is a relatively new machine learning algorithm being used for yield prediction. ENR combines the properties of ridge regression and LASSO (Ogutu et al., 2012), both of which have been successfully applied to crop yield prediction (Kang et al., 2021; Shafiee et al., 2021). The incorporation of multiple VIs adds collinearity to the models, and the ENR is robust to severe multicollinearity among the input features (Ogutu et al., 2012). Another reason for using ENR was the simplicity of the linear model compared to other machine learning algorithms such as RF or ANNs, which makes the model run less time-consuming and more efficient to train.

Several VIs such as normalized difference vegetation index (NDVI) and green normalized difference vegetation index (GNDVI) have been evaluated to monitor crop health under stress and predict the GY. Most of the multispectral VIs that have been reported were species-specific and easily saturated (Hatfield and Prueger, 2013). Therefore, it is challenging to predict important crop traits using a single VI (Wang et al., 2016). The successful use of multiple VIs to improve the prediction accuracy of important traits in crops has been reported in many studies (Wang et al., 2016; Jin et al., 2020). In this study, 21 multispectral VIs and 1 temperature index (i.e., NRCT) were measured in multiple growth stages to validate the UAV data and ENR and check their accuracy for GY prediction. For accurate yield predictions and to avoid model overfitting, the machine learning algorithms may benefit from using a feature selection algorithm to reduce the dimensionality of the data to an appropriate level (Yoosefzadeh-Najafabadi et al., 2021). The GRA is a widely accepted approach in feature selection (Deris et al., 2013; Lu et al., 2019; Yao et al., 2019; Miswan et al., 2021). The results in this study show that GRA can reduce the dimensionality of the input features to some extent.

The model performed poorly when using multispectral VIs to predict yield. This could be due to the saturation issue associated with the visible-near-infrared (Vis-NIR) sensor for dense vegetation such as wheat, soybean, and rice (Thenkabail et al., 2000; Tilly et al., 2015). The fusion of multispectral and thermal features includes canopy spectral and temperature information outperformed for yield prediction, which was consistent with previous reports (Maimaitijiang et al., 2017, 2020). Temperature is closely related to plant physiological processes such as transpiration, leaf water potential, and photosynthesis (Sagan et al., 2019). Generally, high canopy temperature is negatively correlated with crop yield (Tattaris et al., 2016; Sagan et al., 2019). Previously, the UAV-based thermal IR data has been successfully applied to plant trait evaluation (Gonzalez-Dugo et al., 2013; Ludovisi et al., 2017; Liu et al., 2018; Raeva et al., 2019; Crusiol et al., 2020). Previous studies have also shown that combining thermal data with multispectral data outperformed as compared to the fusion of spectral and structural information from RGB and multispectral images for the prediction of LAI, biomass, chlorophyll, and nitrogen in soybean (Maimaitijiang et al., 2017).

The spatial heterogeneity of the ground changes among the developmental stages of the crop could lead to a significant difference in the prediction accuracy across the growth stages (Juliane et al., 2014). The results of yield prediction based on the individual growth stages are similar to previous reports, i.e., wheat yield prediction accuracy was higher at grain filling stages under different growth environments (Hernandez et al., 2015; Hassan et al., 2019a). During the grain filling stage, the starch, protein, and organic matter produced by photosynthesis are transported to the grain (Guan et al., 2017), and this stage is closely linked to the thousand-grain weight. Therefore, the accuracy of yield estimation was highest at the grain filling stage. In addition, the reduction in the greenness and chlorophyll level after the grain filling stages due to the decrease in the degree of dry matter accumulation in the leaves of plants could influence the detection accuracy of VIs based on the red and near-IR light (Yue et al., 2017). This reduces the model performance in the late developmental stage, which causes a decrease in yield prediction accuracy at maturity. In addition, crop canopy information at varying growth stages is associated with different yield elements, and a combination of remote sensing data from multiple growth stages can effectively improve the yield prediction accuracy.

Another main objective of this study was to use an appropriate method to acquire the prediction values from a combination of temporal remote sensing data across the growth cycle. Although previous studies used temporal VIs for yield prediction, most of them used a single VI (Wang et al., 2014; Zhou et al., 2017), which can be influenced by different degrees of saturability or soil background (Wang et al., 2016). We first directly used the multispectral and thermal features from multiple stages as inputs to ENR, and this method was able to obtain higher yield prediction accuracy than individual stages, but the accuracy parameters fluctuated more compared to EMF and were slightly lower than the prediction accuracy of the EMF method for some combinations. This may be due to the redundant information generated by the accumulation of features from multiple growth stages. In addition, the excessive dimensionality of input features also poses the risk of overfitting the machine learning model (Feng et al., 2017; Coolen et al., 2020). Among the combinations of EMF, the prediction accuracy of C2 was comparable to a combination with the highest prediction accuracy of C4. The data required for C2 can be obtained at the flowering stage, which is appropriate for application in practical management. The results of this study suggest that the fusion of multispectral and thermal features within an entropy weight ensemble framework can provide accurate wheat yield predictions. However, more comprehensive studies, such as studies of different crop varieties in different environments, are needed to determine the most accurate and efficient multistage data for combination.

## Conclusion

A rapid and nondestructive method for an accurate GY prediction of wheat is desired in breeding programs. In this study, an ensemble framework was developed to increase the GY prediction accuracy by integrating the predicted values from multiple stages using the UAV-based multispectral and thermal IR imagery. The test results showed that the prediction accuracy of the grain filling stage was the highest among the six growth stages. The ensemble method outperformed the individual stage-based GY prediction in terms of accuracy. Combining the features of the first four growth stages allows for early and accurate yield prediction to aid in decision-making. This study offers a new method for GY prediction through UAV-based remote sensing, and it can help in large breeding activities.

## Data Availability Statement

The original contributions presented in the study are included in the article/supplementary material, further inquiries can be directed to the corresponding author/s.

## Author Contributions

SF analyzed the data and wrote the manuscript. ZC and YX directed the trial and provided the main idea. QC and ZL helped to collect data. MH, YM, and MS provided comments and suggestions to improve the manuscript. All authors read and agreed to the published version of the manuscript.

## Conflict of Interest

The authors declare that the research was conducted in the absence of any commercial or financial relationships that could be construed as a potential conflict of interest. The reviewer AR declared a past co-authorship with the authors MH, YX to the handling editor.

## Publisher’s Note

All claims expressed in this article are solely those of the authors and do not necessarily represent those of their affiliated organizations, or those of the publisher, the editors and the reviewers. Any product that may be evaluated in this article, or claim that may be made by its manufacturer, is not guaranteed or endorsed by the publisher.

## Footnotes

1
https://www.qgis.org/

2
https://CRAN.R-project.org/package=caret

## References

[B1] Appeltans S. Guerrero A. Nawar S. Pieters J. Mouazen A. M. (2020). Practical recommendations for hyperspectral and thermal proximal disease sensing in potato and leek fields. *Remote Sens.* 12:1939. 10.3390/rs12121939

[B2] Aslan N. Shahrivar A. A. Abdollahi H. (2012). Multi-objective optimization of some process parameters of a lab-scale thickener using gray relational analysis. *Sep. Purif. Technol.* 90 189–195. 10.1016/j.seppur.2012.02.033

[B3] Ba Ret F. Guyot G. (1991). Potentials and limits of vegetation indices for LAI and APAR assessment. *Remote Sens. Environ.* 35 161–173. 10.1016/0034-4257(91)90009-U

[B4] Bradley J. B. (1995). Neural networks: A comprehensive foundation. *Inf. Process. Manage.* 31 786. 10.1016/0306-4573(95)90003-9

[B5] Breiman L. (2001). Random forest. *Mach. Learn.* 45 5–32. 10.1023/a:101093340432

[B6] Broge N. H. Leblanc E. (2001). Comparing prediction power and stability of broadband and hyperspectral vegetation indices for estimation of green leaf area index and canopy chlorophyll density. *Remote Sens. Environ.* 76 156–172. 10.1016/S0034-4257(00)00197-8

[B7] Chen J. M. (1996). Evaluation of vegetation indices and a modified simple ratio for boreal applications. *Can. J. Remote. Sens.* 22 229–242. 10.1080/07038992.1996.10855178

[B8] Cheng W. Xi H. Sindikubwabo C. Si J. Wu T. (2020). Ecosystem health assessment of desert nature reserve with entropy weight and fuzzy mathematics methods: A case study of Badain Jaran Desert. *Ecol. Indic.* 119:106843. 10.1016/j.ecolind.2020.106843

[B9] Colomina I. Molina P. (2014). Unmanned aerial systems for photogrammetry and remote sensing: A review. ISPRS J. Photogramm. *Remote Sens.* 92 79–97. 10.1016/j.isprsjprs.2014.02.013

[B10] Comba L. Biglia A. Aimonino D. R. Tortia C. Gay P. (2020). Leaf Area Index evaluation in vineyards using 3D point clouds from UAV imagery. *Precis. Agric.* 21 881–896. 10.1007/s11119-019-09699-x

[B11] Coolen A. C. C. Sheikh M. Mozeika A. Aguirre-Lopez F. Antenucci F. (2020). Replica analysis of overfitting in generalized linear regression models. *J. Phys. A Math. Theor.* 53 10.1088/1751-8121/aba028

[B12] Crusiol L. G. T. Nanni M. R. Furlanetto R. H. Sibaldelli R. N. R. Cezar E. Mertz-Henning L. M. (2020). UAV-based thermal imaging in the assessment of water status of soybean plants. *Int. J. Remote Sens.* 41 3243–3265. 10.1080/01431161.2019.1673914

[B13] Dash J. Curran P. J. (2004). “Evaluation of the meris terrestrial chlorophyll index” in *Proceedings of the IEEE International Geoscience and Remote Sensing Symposium (IGARSS).* (United States: IEEE). 1–257.

[B14] Daughtry C. Walthall C. L. Kim M. S. Colstoun E. McMurtrey M. M. III. (2000). Estimating corn leaf chlorophyll concentration from leaf and canopy reflectance. *Remote Sens. Environ.* 74 229–239. 10.1016/S0034-4257(00)00113-9

[B15] Deris A. M. Zain A. M. Sallehuddin R. (2013). Hybrid GR-SVM for prediction of surface roughness in abrasive water jet machining. *Meccanica* 48 1937–1945. 10.1007/s11012-013-9710-2

[B16] Diker K. Bausch W. C. (2003). Potential use of nitrogen reflectance index to estimate plant parameters and yield of maize. *Biosyst. Eng.* 85 437–447. 10.1016/S1537-5110(03)00097-7

[B17] Eitel J. U. H. Long D. S. Gessler P. E. Smith A. M. S. (2007). Using in-situ measurements to evaluate the new RapidEye™ satellite series for prediction of wheat nitrogen status. *Int. J. Remote Sens.* 28 4183–4190. 10.1080/01431160701422213

[B18] Elmetwalli A. H. El-Hendawy S. Al-Suhaibani N. Alotaibi M. Tahir M. U. Mubushar M. (2020). Potential of hyperspectral and thermal proximal sensing for estimating growth performance and yield of soybean exposed to different drip irrigation regimes under arid conditions. *Sensors.* 20:6569. 10.3390/s20226569 33213009 PMC7698533

[B19] Elsayed S. Elhoweity M. Ibrahim H. H. Dewir Y. H. Migdadi H. M. Schmidhalter U. (2017). Thermal imaging and passive reflectance sensing to estimate the water status and grain yield of wheat under different irrigation regimes. *Agric. Water Manag.* 189 98–110. 10.1016/j.agwat.2017.05.001

[B20] Elsayed S. Rischbeck P. Schmidhalter U. (2015). Comparing the performance of active and passive reflectance sensors to assess the normalized relative canopy temperature and grain yield of drought-stressed barley cultivars. *Field Crop. Res.* 177 148–160. 10.1016/j.fcr.2015.03.010

[B21] Farhadinia B. (2017). A multiple criteria decision making model with entropy weight in an interval-transformed hesitant fuzzy environment. *Cogn Comput.* 9 513–525. 10.1007/s12559-017-9480-6

[B22] Fei S. Hassan M. A. He Z. Chen Z. Shu M. Wang J. (2021). Assessment of ensemble learning to predict wheat grain yield based on UAV-Multispectral reflectance. *Remote Sens.* 13:2338. 10.3390/rs13122338

[B23] Feng X. Liang Y. Shi X. Xu D. Wang X. Guan R. (2017). Overfitting reduction of text classification based on AdaBELM. *Entropy* 19:330. 10.3390/e19070330

[B24] Gitelson A. A. Kaufman Y. J. Merzlyak M. N. (1996). Use of a green channel in remote sensing of global vegetation from EOS-MODIS. *Remote Sens. Environ.* 58 289–298. 10.1016/S0034-4257(96)00072-7

[B25] Gitelson A. A. Vina A. Arkebauer T. J. Rundquist D. C. Keydan G. Leavitt B. (2003). Remote estimation of leaf area index and green leaf biomass in maize canopies. *Geophys. Res. Lett.* 30:1248. 10.1029/2002GL016450

[B26] Goel N. S. Qin W. (1994). Influences of canopy architecture on relationships between various vegetation indices and LAI and Fpar: A computer simulation. *Remote Sens. Environ.* 10 309–347. 10.1080/02757259409532252

[B27] Gonzalez-Dugo V. Zarco-Tejada P. Nicolás E. Nortes P. A. Alarcón J. J. Intrigliolo D. S. (2013). Using high resolution UAV thermal imagery to assess the variability in the water status of five fruit tree species within a commercial orchard. *Precis. Agric.* 14 660–678. 10.1007/s11119-013-9322-9

[B28] Guan K. Wu J. Kimball J. S. Anderson M. C. Frolking S. Li B. (2017). The shared and unique values of optical, fluorescence, thermal and microwave satellite data for estimating large-scale crop yields. *Remote Sens. Environ.* 199 333–349. 10.1016/j.rse.2017.06.043

[B29] Haboudane D. Miller J. R. Pattey E. Zarco-Tejada P. J. Strachan I. B. (2004). Hyperspectral vegetation indices and novel algorithms for predicting green LAI of crop canopies: Modeling and validation in the context of precision agriculture. *Remote Sens. Environ.* 90 337–352. 10.1016/j.rse.2003.12.013

[B30] Hassan M. A. Yang M. Awais R. Jin X. Xia X. Xiao Y. (2018). Time-series multispectral indices from unmanned aerial vehicle imagery reveal senescence rate in bread wheat. *Remote Sens.* 10:809. 10.3390/rs10060809

[B31] Hassan M. A. Yang M. Awais R. Yang G. Reynolds M. Xia X. (2019a). A rapid monitoring of NDVI across the wheat growth cycle for grain yield prediction using a multi-spectral UAV platform. *Plant Sci.* 282 95–103. 10.1016/j.plantsci.2018.10.022 31003615

[B32] Hassan M. A. Yang M. Fu L. Rasheed A. Zheng B. Xia X. (2019b). Accuracy assessment of plant height using an unmanned aerial vehicle for quantitative genomic analysis in bread wheat. *Plant Methods* 15:37. 10.1186/s13007-019-0419-7 PMC6463666 31011362

[B33] Hassan M. A. Yang M. Rasheed A. Tian X. Reynolds M. Xia X. (2021). Quantifying senescence in bread wheat using multispectral imaging from an unmanned aerial vehicle and QTL mapping. *Plant Phys.* 2021 kiab 431, 10.1093/plphys/kiab431 PMC8644761 34601616

[B34] Hatfield J. L. Prueger J. H. (2013). Value of using different vegetative indices to quantify agricultural crop characteristics at different growth stages under varying management practices. *Remote Sens.* 2 562–578. 10.3390/rs2020562

[B35] Hernandez J. Lobos G. A. Matus I. Del Pozo A. Silva P. Galleguillos M. (2015). Using ridge regression models to estimate grain yield from field spectral data in bread wheat (Triticum Aestivum L.) grown under three water regimes. *Remote Sens.* 7 2109–2126. 10.3390/rs70202109

[B36] Huete A. Didan K. Miura T. Rodriguez E. P. Gao X. Ferreira L. G. (2002). Overview of the radiometric and biophysical performance of the MODIS vegetation indices. *Remote Sens. Environ.* 83 195–213. 10.1016/S0034-4257(02)00096-2

[B37] Huete A. R. (1988). A soil-adjusted vegetation index (SAVI). *Remote Sens. Environ.* 25 295–309. 10.1016/0034-4257(88)90106-X

[B38] Hui Z. Hastie T. (2005). Regularization and variable selection via the elastic net. *J. R. Statist. Soc. B* 67:768. 10.1111/j.1467-9868.2005.00527.x

[B39] Jin X. Li Z. Feng H. Ren Z. Li S. (2020). Deep neural network algorithm for estimating maize biomass based on simulated Sentinel 2A vegetation indices and leaf area index. *Crop J.* 8 87–97. 10.1016/j.cj.2019.06.005

[B40] Jin X. Xu X. Song X. Li Z. Wang J. Guo W. (2013). Estimation of leaf water content in winter wheat using gray relational analysis–partial least squares modeling with hyperspectral data. *Agron. J.* 105 1385–1392. 10.2134/agronj2013.0088

[B41] Juliane B. Andreas B. Simon B. Janis B. Silas E. Georg B. (2014). Estimating biomass of barley using crop surface models (CSMs) derived from UAV-based RGB imaging. *Remote Sens.* 6 10395–10412. 10.3390/rs61110395

[B42] Kang Y. Nam J. Kim Y. Lee S. Seong D. Jang S. (2021). Assessment of regression models for predicting rice yield and protein content using unmanned aerial Vehicle-Based multispectral imagery. *Remote Sens.* 13:1508. 10.3390/rs13081508

[B43] Li H. Li D. Li Y. (2018). A multi-index assessment method for evaluating coverage effectiveness of remote sensing satellite. *Chinese J. Aeronaut.* 151 98–108. 10.1016/j.cja.2018.05.015

[B44] Li L. Zhang Q. Huang D. (2014). A review of imaging techniques for plant phenotyping. *Sensors.* 14 20078–20111. 10.3390/s141120078 25347588 PMC4279472

[B45] Li X. Wang K. Liu L. Jing X. Yang H. Gao C. (2011). Application of the entropy weight and topsis method in safety evaluation of coal mines. *Procedia Eng.* 26 2085–2091. 10.1016/j.proeng.2011.11.2410

[B46] Liu H. Zhang X. Xu Y. Ma F. Zhang J. Cao Y. (2020). Identification and validation of quantitative trait loci for kernel traits in common wheat (Triticum Aestivum L.). *BMC Plant Biol.* 20:529. 10.1186/s12870-020-02661-4 33225903 PMC7682089

[B47] Liu T. Li R. Zhong X. Jiang M. Jin X. Zhou P. (2018). Estimates of rice lodging using indices derived from UAV visible and thermal infrared images. *Agr. Forest Meteorol.* 252 144–154. 10.1016/j.agrformet.2018.01.021

[B48] Lu D. Yu H. L. Yu G. M. (2014). Assessing the land use change and ecological security based on RS and GIS: A case study of Pingdingshan city. *China. Adv. Mater. Res.* 905 329–333. 10.4028/www.scientific.net/AMR.905.329

[B49] Lu S. X. Lin G. Que H. Li M. J. J. Wei C. H. Wang J. K. (2019). Grey relational analysis using Gaussian process regression method for dissolved gas concentration prediction. *Int J Mach Learn Cyb.* 10 1313–1322. 10.1007/s13042-018-0812-y

[B50] Ludovisi R. Tauro F. Salvati R. Khoury S. Harfouche A. (2017). UAV-based thermal imaging for high-throughput field phenotyping of black poplar response to drought. *Front. Plant Sci.* 8:1681. 10.3389/fpls.2017.01681 29021803 PMC5623950

[B51] Maimaitijiang M. Ghulam A. Sidike P. Hartling S. Maimaitiyiming M. Peterson K. (2017). Unmanned aerial system (UAS)-based phenotyping of soybean using multi-sensor data fusion and extreme learning machine. *ISPRS J. Photogramm. Remote Sens.* 134 43–58. 10.1016/j.isprsjprs.2017.10.011

[B52] Maimaitijiang M. Sagan V. Sidike P. Hartling S. Esposito F. Fritschi F. B. (2020). Soybean yield prediction from UAV using multimodal data fusion and deep learning. *Remote Sens. Environ.* 237:111599. 10.1016/j.rse.2019.111599

[B53] Mcbratney A. Whelan B. Ancev T. Bouma J. (2005). Future directions of precision agriculture. *Precis. Agric.* 6 7–23. 10.1007/s11119-005-0681-8

[B54] Merzlyak M. N. Gitelson A. A. Chivkunova O. B. Rakitin V. Y. (1999). Non-destructive optical detection of pigment changes during leaf senescence and fruit ripening. *Physiol. Plant.* 106 135–141. 10.1034/j.1399-3054.1999.106119.x 11841302

[B55] Metternicht G. (2003). Vegetation indices derived from high-resolution airborne videography for precision crop management. *Int. J. Remote Sens.* 24 2855–2877. 10.1080/01431160210163074

[B56] Miswan N. H. Chan C. S. Ng C. G. (2021). *Hospital Readmission Prediction Based on Improved Feature Selection Using Grey Relational Analysis and LASSO.* United Kingdom: Emerald Group Publishing. 10.1108/GS-12-2020-0168

[B57] Ogutu J. O. Schulz-Streeck T. Piepho H. (2012). Genomic selection using regularized linear regression models: ridge regression, lasso, elastic net and their extensions. *BMC Proc.* 6:S10. 10.1186/1753-6561-6-S2-S10 22640436 PMC3363152

[B58] Osval A. Montesinos López A. M. L. José C. Gustavo D. L. C. Alvarado G. Mondal S. (2017). Predicting grain yield using canopy hyperspectral reflectance in wheat breeding data. *Plant Methods* 13:4. 10.1186/s13007 PMC5209864 28053649

[B59] Panda S. S. Ames D. P. Panigrahi S. (2010). Application of vegetation indices for agricultural crop yield prediction using neural network techniques. *Remote Sens.* 2:673. 10.3390/rs2030673

[B60] Penuelas J. Filella I. Gamon J. A. (1995). Assessment of photosynthetic radiation-use efficiency with spectral reflectance. *New Phytol.* 131 291–296. 10.1111/j.1469-8137.1995.tb03064.x

[B61] Qiao L. Gao D. Zhang J. Li M. Ma J. (2020). Dynamic influence elimination and chlorophyll content diagnosis of maize using UAV spectral imagery. *Remote Sens.* 12:2650. 10.3390/rs12162650

[B62] Raeva P. L. Šedina J. Dlesk A. (2019). Monitoring of crop fields using multispectral and thermal imagery from UAV. *Eur. J. Remote Sens.* 52 192–201. 10.1080/22797254.2018.1527661

[B63] Rischbeck P. Baresel P. Elsayed S. Mistele B. Schmidhalter U. (2014). Development of a diurnal dehydration index for spring barley phenotyping. *Funct. Plant Biol.* 41 1249–1260. 10.1071/FP14069 32481074

[B64] Rondeaux G. Steven M. Baret F. (1996). Optimization of soil-adjusted vegetation indices. *Remote Sens. Environ.* 55 95–107. 10.1016/0034-4257(95)00186-7

[B65] Rouse J. Jr. (1972). “Monitoring the vernal advancement and retrogradation (green WaveEffect) of natural vegetation” in *Nasa/Gsfct Type Final Report.* (Maryland: NASA Goddard Space Flight Center).

[B66] Sagan V. Maimaitijiang M. Sidike P. Eblimit K. Peterson K. Hartling S. (2019). UAV-based high resolution thermal imaging for vegetation monitoring, and plant phenotyping using ICI 8640 P, FLIR Vue Pro R 640, and thermoMap Cameras. *Remote Sens.* 11:330. 10.3390/rs11030330

[B67] Sain S. R. (1997). The nature of statistical learning theory. *Technometrics* 38:409. 10.1080/00401706.1996.10484565

[B68] Shafiee S. Lied L. M. Burud I. Dieseth J. A. Alsheikh M. Lillemo M. (2021). Sequential forward selection and support vector regression in comparison to LASSO regression for spring wheat yield prediction based on UAV imagery. *Comput. Electron. Agr.* 183:106036. 10.1016/j.compag.2021.106036

[B69] Shah S. H. Angel Y. Houborg R. Ali S. McCabe M. F. (2019). A random forest machine learning approach for the retrieval of leaf chlorophyll content in wheat. *Remote Sens.* 11:920. 10.3390/rs11080920

[B70] Sidike P. Sagan V. Qumsiyeh M. Maimaitijiang M. Essa A. Asari V. (2018). *Adaptive Trigonometric Transformation Function with Image Contrast and Color Enhancement: application to Unmanned Aerial System Imagery.* United Kingdom: IEEE. 1–5. 10.1109/LGRS.2018.2790899

[B71] Suyoung P. Dongryeol R. Sigfredo F. Hoam C. Esther H. M. Mark O. (2017). Adaptive estimation of crop water stress in nectarine and peach orchards using high-resolution imagery from an unmanned aerial vehicle (UAV). *Remote Sens.* 9:828. 10.3390/rs9080828

[B72] Tattaris M. Reynolds M. P. Chapman S. C. (2016). A direct comparison of remote sensing approaches for high-throughput phenotyping in plant breeding. *Front. Plant Sci.* 7:1131. 10.3389/fpls.2016.01131 27536304 PMC4971441

[B73] Tavakoli H. Gebbers R. (2019). Assessing nitrogen and water status of winter wheat using a digital camera. *Comput. Electron. Agr.* 157 558–567. 10.1016/j.compag.2019.01.030

[B74] Thenkabail P. S. Smith R. B. De Pauw E. (2000). Hyperspectral vegetation indices and their relationships with agricultural crop characteristics. *Remote Sens. Environ.* 71 158–182.

[B75] Tilly N. Aasen H. Bareth G. (2015). Fusion of plant height and vegetation indices for the estimation of barley biomass. *Remote Sens.* 7 17291–17296.

[B76] Tucker C. J. Elgin J. H. McMurtrey M. M. III. Fan C. J. (1979). Monitoring corn and soybean crop development with hand-held radiometer spectral data. *Remote Sens. Environ.* 8 237–248. 10.1016/0034-4257(79)90004-X

[B77] Wang J. Chen Y. Chen F. Shi T. Wu G. (2018). Wavelet-based coupling of leaf and canopy reflectance spectra to improve the estimation accuracy of foliar nitrogen concentration. *Agr. Forest Meteorol.* 248 306–315. 10.1016/j.agrformet.2017.10.017

[B78] Wang L. Chang Q. Yang J. Zhang X. Li F. Xu Y. (2018). Estimation of paddy rice leaf area index using machine learning methods based on hyperspectral data from multi-year experiments. *PLoS One* 13:e0207624. 10.1371/journal.pone.0207624 30517144 PMC6281281

[B79] Wang K. Shen Z. Q. Wang R. C. (1998). Effects of nitrogen nutrition on the spectral reflectance characteristics of rice leaf and canopy. *J. Zhejiang Agric. Univ.* 24 93–97.

[B80] Wang L. Tian Y. Yao X. Zhu Y. Cao W. (2014). Predicting grain yield and protein content in wheat by fusing multi-sensor and multi-temporal remote-sensing images. *Field Crop. Res.* 164 178–188. 10.1016/j.fcr.2014.05.001

[B81] Wang L. Zhou X. Zhu X. Dong Z. Guo W. (2016). Estimation of biomass in wheat using random forest regression algorithm and remote sensing data. *Crop J.* 4 212–219. 10.1016/j.cj.2016.01.008

[B82] Yang M. Hassan M. A. Xu K. Zheng C. Rasheed A. Zhang Y. (2020). Assessment of water and nitrogen use efficiencies through UAV-based multispectral phenotyping in winter wheat. *Front. Plant Sci.* 11:927. 10.3389/fpls.2020.00927 32676089 PMC7333459

[B83] Yao Z. Zhang T. Wang J. Zhu L. (2019). A feature selection approach based on grey relational analysis for within-project software defect prediction. *J. Grey Syst.* 31 105–116.

[B84] Yoosefzadeh-Najafabadi M. Earl H. J. Tulpan D. Sulik J. Eskandari M. (2021). Application of machine learning algorithms in plant breeding: Predicting yield from hyperspectral reflectance in soybean. *Front. Plant Sci.* 11:624273. 10.3389/fpls.2020.624273 33510761 PMC7835636

[B85] Yu N. Li L. Schmitz N. Tiaz L. F. Greenberg J. A. Diers B. W. (2016). Development of methods to improve soybean yield estimation and predict plant maturity with an unmanned aerial vehicle based platform. *Remote Sens. Environ.* 187 91–101. 10.1016/j.rse.2016.10.005

[B86] Yue J. Yang G. Li C. Li Z. Wang Y. Feng H. (2017). Estimation of winter wheat above-ground biomass using unmanned aerial vehicle-based snapshot hyperspectral sensor and crop height improved models. *Remote Sens.* 9:708. 10.3390/rs9070708

[B87] Yue J. Yang G. Tian Q. Feng H. Xu K. Zhou C. (2019). Estimate of winter-wheat above-ground biomass based on UAV ultrahigh-ground-resolution image textures and vegetation indices. *ISPRS J. Photogramm. Remote Sens.* 150 226–244. 10.1016/j.isprsjprs.2019.02.022

[B88] Zhang L. Zhang Z. Luo Y. Cao J. Tao F. (2020). Combining optical, fluorescence, thermal satellite, and environmental data to predict county-level maize yield in china using machine learning approaches. *Remote Sens.* 12:21. 10.3390/rs12010021

[B89] Zhou X. Zheng H. B. Xu X. Q. He J. Y. Ge X. K. Yao X. (2017). Predicting grain yield in rice using multi-temporal vegetation indices from UAV-based multispectral and digital imagery. *ISPRS J. Photogramm. Remote Sens.* 130 246–255. 10.1016/j.isprsjprs.2017.05.003

[B90] Zubler A. V. Yoon J. (2020). Proximal methods for plant stress detection using optical sensors and machine learning. *Biosensors* 10:193. 10.3390/bios10120193 33260412 PMC7760370

